# A Liquid-Surface-Based Three-Axis Inclination Sensor for Measurement of Stage Tilt Motions

**DOI:** 10.3390/s18020398

**Published:** 2018-01-30

**Authors:** Yuki Shimizu, Satoshi Kataoka, Tatsuya Ishikawa, Yuan-Liu Chen, Xiuguo Chen, Hiraku Matsukuma, Wei Gao

**Affiliations:** Department of Finemechanics, Tohoku University, Sendai 980-8579, Japan; kataoka@nano.mech.tohoku.ac.jp (S.K.); sr-tech@nano.mech.tohoku.ac.jp (T.I.); yuanliuchen@nano.mech.tohoku.ac.jp (Y.-L.C.); xiuguochen@nano.mech.tohoku.ac.jp (X.C.); hiraku.matsukuma@nano.mech.tohoku.ac.jp (H.M.); gaowei@cc.mech.tohoku.ac.jp (W.G.)

**Keywords:** three-axis measurement, inclination, linear slide, optical angle sensor, laser autocollimation

## Abstract

In this paper a new concept of a liquid-surface-based three-axis inclination sensor for evaluation of angular error motion of a precision linear slide, which is often used in the field of precision engineering such as ultra-precision machine tools, coordinate measuring machines (CMMs) and so on, is proposed. In the liquid-surface-based three-axis inclination sensor, a reference float mounting a line scale grating having periodic line grating structures is made to float over a liquid surface, while its three-axis angular motion is measured by using an optical sensor head based on the three-axis laser autocollimation capable of measuring three-axis angular motion of the scale grating. As the first step of research, in this paper, theoretical analysis on the angular motion of the reference float about each axis has been carried out based on simplified kinematic models to evaluate the possibility of realizing the proposed concept of a three-axis inclination sensor. In addition, based on the theoretical analyses results, a prototype three-axis inclination sensor has been designed and developed. Through some basic experiments with the prototype, the possibility of simultaneous three-axis inclination measurement by the proposed concept has been verified.

## 1. Introduction

A precision linear slide is one of the most important components in precision machine tools and precision measuring instruments [1]. With the enhancement of precision positioning sensors such as laser interferometers or linear encoders, a precision linear slide can realize highly accurate and precise positioning [2]. Meanwhile, in recent years, it has become more important to verify the angular error motion of a linear slide quantitatively to achieve further higher positioning accuracy [3].

For evaluation of the tilt angles of a precision linear slide, autocollimators and electronic levels have often been employed so far as traditional optical measuring instruments [4,5]. By employing two-degree-of-freedom position detectors such as CCDs [6] or multi-cell photodiodes [7], simultaneous two-degree-of-freedom measurement of tilt angles of a linear slide can be carried out in a simple manner just by placing a target reflector on a slide table and adjusting the optical axis of an autocollimator with respect to the target. When a two-axis autocollimator is placed in such a way that its optical axis coincides with a primary motion axis of a linear slide, both pitch and yaw angles of the linear slide can be measured simultaneously. Meanwhile, a roll angle, which could significantly affect the positioning accuracy of a linear slide when the measurement axis of a displacement sensor is placed with an offset from the motion axis of the linear slide [2], cannot be measured. For measurement of the roll angle, an additional autocollimator should therefore be employed. However, this also induces another problem; for measurement of the roll angle of a linear slide, a reference mirror with the length covering the long travel range of the linear slide should be employed. Although a laser interferometer system can be employed instead of an autocollimator to evaluate the roll angle of a linear slide having a long travel range [8,9], the measured information will be lost and the evaluation has to be re-started from the beginning if the optical path of the laser interferometer is blocked; this could be a fatal problem in the case of production process of the slide, where the measurement will repeatedly be carried out together with a hand scraping operation for correction of the form errors of slide guideways [10]. Therefore, angle sensors/inclination sensors that can be mounted on a slide table and can carry out highly-accurate simultaneous tilt angle measurement about the *X*-, *Y*- and *Z*-axes are desired to be realized. Gyroscopes such as ring laser gyroscopes [11], fibre optic gyroscopes [12] and vibrating structure gyroscopes [13] could also be one of the candidate instruments satisfying the requirements mentioned above [14]. By utilizing a gyro for each measurement axis, simultaneous measurement of three-axis tilt angles can be realized. Especially, ring laser gyroscopes [11] have a high enough measurement resolution to be applied for the evaluation of tilt angles of a precision linear slide. However, due to the principle of gyroscope in which the tilt angle will be acquired by integrating detected angular velocity, even a high precision ring laser gyroscope could have a bias instability on the order of 0.01°/h (which roughly corresponds to 1 arc-second/100 s) [15]; which cannot be neglected for the evaluation of the angular error motion of a precision linear slide, which is typically on the order of several arc-seconds. In addition, a high cost of the ring laser gyro could also be an obstacle to employ it for the evaluation of linear slides in a machine shop.

The authors have developed an optical angle sensor referred to as the three-axis laser autocollimator [16] that can carry out simultaneous measurement of three-axis tilt angles by using a single laser beam. By employing a line scale grating having periodic line grating structures as a reflector, instead of a flat mirror reflector employed in traditional one-axis or two-axis autocollimators based on the laser autocollimation [17], simultaneous measurement of three-axis tilt angles with a resolution better than one arc second has been realized [16]. In the three-axis laser autocollimator, both the zeroth-order and a first-order diffracted beam from the scale grating are required to be detected by an optical sensor head. Meanwhile, the orientation of the first-order diffracted beam from the scale grating is different from the axis of the measurement beam from the laser autocollimator. The working distance of the three-axis laser autocollimator is therefore limited by the allowable size of the optical sensor head, resulting in the difficulty of evaluating a linear slide having a long stroke. In responding to the background described above, the authors have also developed a high-resolution inclination sensor [18] based on the fluid-based type commercial electronic level [19], which does not require a reflector because its angle reference is the level of liquid enclosed inside of the sensor body [20,21]. One of the main features of the high-resolution inclination sensor is its compact size and simple procedure for the measurement of the roll angle of a slide table in a long-stroke linear slide; this is a great advantage from the viewpoint of its usage in the linear slide production line. It should be noted that the concept of using liquids as reference for flatness and a constant level tilt has long been used for measurement of the pitch or roll angles of a linear slide [22]. Two-axis electronic levels based on this concept that can make simultaneous two-axis measurement of pitch and roll angles are also commercially available [23]. However, a conventional electronic level cannot measure the yaw angle of a linear slide in principle, which is a major drawback compared with the autocollimators and laser interferometers. Although the three-axis tilt angle measurement of a linear slide can be realized by the combination of an electronic level and an autocollimator, this will increase the cost and complicity of the measurement system. Meanwhile, in the case of measurement of a linear slide having long travel range, a long optical path of autocollimator could degrade the measurement accuracy of yaw measurement due to the external disturbances such as temperature deviation in a machine shop. A new type of inclination sensor capable of carrying out simultaneous three-axis tilt angles, not only the pitch and roll angles, but also the yaw angle, is therefore desired for measurement of the tilt angles of a precision linear slide.

In this paper, to achieve simultaneous measurement of three-axis tilt angles of a precision linear slide, a new concept of three-axis inclination sensor is proposed. In the concept, a principle of fluid-based type clinometer is extended by integrating the principle of three-axis laser autocollimator. Simultaneous measurement of three-axis tilt angles can be achieved in such a way that a reference float mounting a scale grating will be made to float over a liquid surface in a casing, while monitoring the tilt angles of the reference float by using an optical sensor head of the three-axis laser autocollimator, which is kinematically connected to the casing. As the first step of research, in this paper, a theoretical analysis on the angular motion of the reference float about each axis based on a simplified kinematic model is at first carried out to search for the possibility of realizing the proposed concept of the three-axis inclination sensor. In addition, based on the results of the theoretical analyses, a prototype three-axis inclination sensor is designed and developed to verify the possibility of simultaneous measurement of three-axis tilt angles by the proposed concept.

## 2. Principle of the Proposed Three-Axis Inclination Sensor

### 2.1. A Concept of the Proposed Three-Axis Inclination Sensor

**Figure 1:**
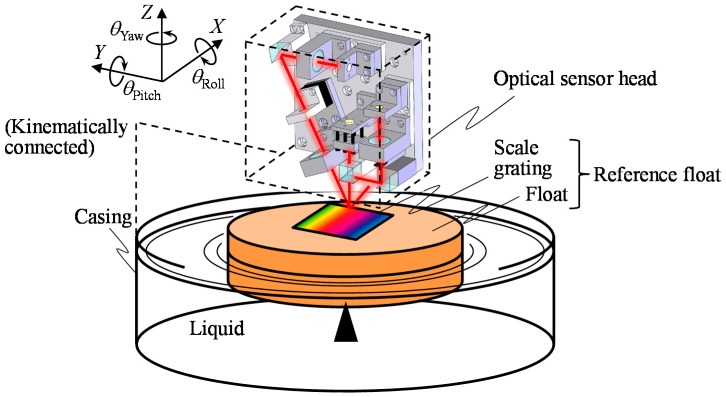
A schematic of the liquid-surface-based three-axis inclination sensor.

A schematic of the proposed concept of the three-axis inclination sensor is shown in Figure 1. The sensor is composed of an optical sensor head and a reference float that floats on a liquid surface in a casing. The optical sensor head is kinematically connected to the casing. The reference float consists of a scale grating with line pattern structures and a float on which the scale grating is mounted. Although similar concepts using liquids as reference for flatness and a constant level tilt can be found in the literature [22], the proposed three-axis inclination sensor is expected to realize simultaneous measurement of rotational motion about the three-axes with the enhancement of three-axis laser autocollimation [16]. The center point in the bottom surface of float is supported by the tip of a tiny needle, which is rigidly fixed to the bottom of the casing, so that the reference float can be held stationary along the *X*-, *Y*- and *Z*-directions in the sensor system, while allowing relative angular motion of the reference float with respect to the casing about each of the three-axes.

Three-axis inclination measurements are carried out by mounting the inclination sensor on a measurement target such as the slide table in a precision linear slide. Since the normal direction of the liquid surface in the casing always coincides with the orientation of gravity, the reference float being floated on the liquid surface can be treated as a datum for tilt angle measurement about the *X*- and *Y*-axes. By detecting relative tilt angles of the optical sensor head with respect to the reference float, tilt angles of the measurement target about the *X*- and *Y*-axes can be measured.

The reference float can also be employed as a datum for tilt angle measurement about the *Z*-axis. Since the reference float is being floated on a liquid surface, an external torque to be applied to the reference float will be generated by shearing force between the float surface and the liquid, as well as by the friction force between the bottom surface of the reference float and a supporting needle introduced to support the reference float. On the assumption that the frictional force due to the supporting needle is small enough to be neglected, the angular motion of the float about the *Z*-axis can be suppressed by employing liquid with low viscosity resistance, and therefore the reference float is expected to be used as a datum for measurement of inclination angle about the *Z*-axis since the angular position of the reference float about the *Z*-axis with respect to a fixed table, where the measurement target is mounted, is expected to be kept stationary with the enhancement of the inertial force of the reference float. It should be noted that, for the evaluation of stage tilt, employing another reference three-axis inclination sensor mounted on a table where the stage is mounted realizes relative angular position measurement with respect to the angular position of the stage at its initial position. In the following section, to verify the feasibility of proposed three-axis inclination sensor, a theoretical analysis on the angular motion of the reference float is carried out based on simplified kinematic models of the float on the liquid surface.

### 2.2. Modeling of the Yaw Motion of Inclination Angle Reference Float

In the proposed three-axis inclination sensor, drift motion of the reference float about the *Z*-axis (yaw drift), which could occur due to the liquid flow around the reference float induced by the angular motion of the casing about the *Z*-axis, degrades the accuracy of yaw angle measurement. The reference float is therefore required to be designed while paying attention to its sensitivity against the liquid flow about the *Z*-axis. The equation of motion of the reference float about the *Z*-axis can be expressed as follows by the second-order differential equation:

(1) $$I_{z}\frac{d^{2}\theta_{\mathrm{Yaw}}\left(t\right)}{dt^{2}}=M_{\mathrm{Ext}}(t)$$

where *θ*_Yaw_ and *I_Z_* are the angular displacement and moment of inertia of the reference float about the *Z*-axis, *M*_Ext_ is external torque applied to the reference float about the *Z*-axis, and *t* is time. Since both *θ*_Yaw_ and *M*_Ext_ are functions of *t*, now we discretize the equation over time interval Δ*t* as follows:

(2) $$I_{z}\frac{\frac{\Delta \theta_{\mathrm{Yaw}}(t_{i})}{\Delta t}-\frac{\Delta \theta_{\mathrm{Yaw}}(t_{i-1})}{\Delta t}}{\Delta t}=M_{\mathrm{Ext}}(t_{i})$$

where *t_i_* (*i* = 1, 2, …, *N*) is discrete time, and Δθ_Yaw_(*t_i_*) = θ_Yaw_(*t_i_*) − θ_Yaw_(*t_i_*_−1_). By solving this equation about Δθ_Yaw_(*t_i_*), the following equation can be obtained:

(3) $$\Delta \theta_{\mathrm{Yaw}}(t_{i})=\Delta \theta_{\mathrm{Yaw}}(t_{i-1})+\frac{M_{\mathrm{Ext}}(t_{i})}{I_{z}}\Delta t^{2}$$

Equation (3) shows that large moment of inertia *I_Z_* of the reference float contributes to reduce the yaw drift, as well as the decrease of the external torque *M*_Ext_. In this paper, the external torque to be applied to the reference float is modeled as a sum of shear torque *M*_Bottom_(*t*) generated by the liquid flow between the bottom surface of the casing and that of the float, and shear torque *M*_Side_(*t*) generated by the liquid flow between the side wall of the casing and that of the reference float.

**Figure 2:**
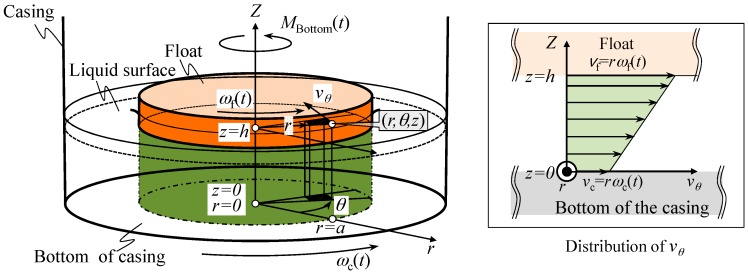
Modeling of the shear torque in between the bottom surface of casing and that of reference float.

At first, *M*_Bottom_(*t*) is estimated based on a simple model shown in Figure 2. Now we assume that the liquid in the casing can be treated as the Newtonian fluid, and it flows only in the circumferential direction about the *Z*-axis in the figure. In this case, the shear stress *τ* in the liquid can be expressed as follows [24]:

(4) $$\tau =\mu \frac{dv}{dz}$$

where *µ* is the shear viscosity of the liquid, and *dv*/*dz* is the derivative of the velocity *v* parallel to the direction of shear relative to the *Z*-directional displacement *z*. 

Denoting the angular velocities of the float and the casing as *ω*_f_(*t*) and *ω*_c_(*t*), respectively, the fluid velocity at the bottom surface of float *v*_f_ and that at the bottom surface of casing *v*_c_ can be expressed as *v*_f_ = *rω_f_*(*t*) and *v*_c_ = *rω*_c_(*t*), respectively. The shear stress *d*τ to be applied to the area element *dS* can therefore be expressed as follows:

(5) $$d\tau =\mu \frac{v_{c}-v_{f}}{h}=\mu r\frac{\omega_{c}(t)-\omega_{f}(t)}{h}$$

Now the area element *dS* in the polar coordinate system can be expressed as *dS* = *rdrdθ*, where *r* and θ are a radial position and an angular position, respectively. The shear torque to be applied to the area element *dS* can therefore be derived as follows:

(6) $$dM_{Bottom}(t)=rd\tau dS=\mu r^{3}\frac{\omega_{c}(t)-\omega_{f}(t)}{h}drd\theta$$

The total shear torque *M*_Bottom_(*t*) to be applied to the bottom surface of the float can be acquired by integrating Equation (6) over the whole bottom surface as follows:

(7) $$M_{Bottom}(t)=\int_{0}^{2\pi }\int_{0}^{a}\mu r^{3}\frac{\omega_{c}(t)-\omega_{f}(t)}{h}drd\theta =\frac{\pi \mu a^{4}}{2h}(\omega_{c}(t)-\omega_{f}(t))$$

where *a* is the radius of the float.

**Figure 3:**
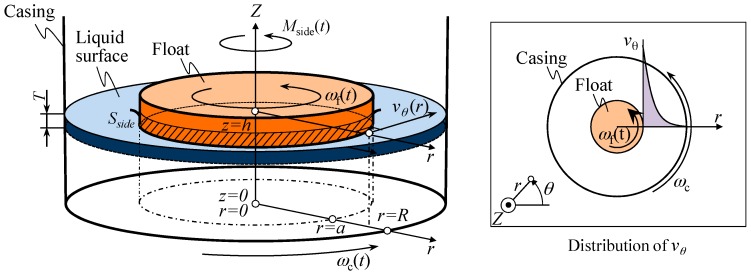
Modeling of the shear torque between the sidewalls of casing and float.

As a next step of the modeling of the yaw motion of the reference float, the shear torque *M*_Side_(*t*) is estimated. Figure 3 shows a schematic of the modeling of shear torque between the sidewall of casing and that of float. In this model, a cylindrical coordinate system is employed to describe the motion of reference float. Denoting the radial and circumferential components of fluid velocity as *v_r_* and *v_θ_*, respectively, shear stress τ*_r__θ_* to be generated by the liquid viscosity can be expressed as follows [24]:

(8) $$\tau_{r\theta }=\mu \left\{r\frac{\partial }{\partial r}\left(\frac{v_{\theta }}{r}\right)+\frac{1}{r}\frac{\partial v_{r}}{\partial \theta }\right\}$$

Assuming *v*_r_ = 0 for the sake of simplicity, the above equation can be simplified as follows:

(9) $$\tau_{r\theta }=\mu r\frac{\partial }{\partial r}\left(\frac{v_{\theta }}{r}\right)$$

The circumferential liquid velocity component *v_θ_* can be obtained by deriving Navier–Stokes equations. Denoting the *Z*-directional liquid velocity component as *v_Z_* and body forces along the radial, circumferential and the *Z*-directions as *F_r_*, *F_θ_* and *F_Z_*, respectively, incompressible momentum Navier-Stokes equations in the cylindrical coordinate system can be expressed as follows [25]:

(10) $$\begin{array}{l} \frac{\partial v_{r}}{\partial t}+v_{r}\frac{\partial v_{r}}{\partial r}+\frac{v_{\theta }}{r}\frac{\partial v_{r}}{\partial \theta }-\frac{{v_{\theta }}^{2}}{r}+v_{z}\frac{\partial v_{r}}{\partial z}\\ \;=-\frac{1}{\rho }\frac{\partial p}{\partial r}+\nu \left(\frac{\partial^{2}v_{r}}{\partial r^{2}}+\frac{1}{r}\frac{\partial v_{r}}{\partial r}-\frac{v_{r}}{r^{2}}+\frac{1}{r^{2}}\frac{\partial^{2}v_{r}}{\partial \theta^{2}}-\frac{2}{r^{2}}\frac{\partial v_{\theta }}{\partial \theta }+\frac{\partial^{2}v_{r}}{\partial z^{2}}\right)+F_{r} \end{array}$$

(11) $$\begin{array}{l} \frac{\partial v_{\theta }}{\partial t}+v_{r}\frac{\partial v_{\theta }}{\partial r}+\frac{v_{\theta }}{r}\frac{\partial v_{\theta }}{\partial \theta }-\frac{v_{r}v_{\theta }}{r}+v_{z}\frac{\partial v_{\theta }}{\partial z}\\ \;=-\frac{1}{\rho }\frac{\partial p}{r\partial \theta }+\nu \left(\frac{\partial^{2}v_{\theta }}{\partial r^{2}}+\frac{1}{r}\frac{\partial v_{\theta }}{\partial r}-\frac{v_{\theta }}{r^{2}}+\frac{1}{r^{2}}\frac{\partial^{2}v_{\theta }}{\partial \theta^{2}}+\frac{2}{r^{2}}\frac{\partial v_{r}}{\partial \theta }+\frac{\partial^{2}v_{\theta }}{\partial z^{2}}\right)+F_{\theta } \end{array}$$

(12) $$\begin{array}{l} \frac{\partial v_{z}}{\partial t}+v_{r}\frac{\partial v_{z}}{\partial r}+\frac{v_{\theta }}{r}\frac{\partial v_{z}}{\partial \theta }+v_{z}\frac{\partial v_{z}}{\partial z}\\ \;=-\frac{1}{\rho }\frac{\partial p}{\partial z}+\nu \left(\frac{\partial^{2}v_{z}}{\partial r^{2}}+\frac{1}{r}\frac{\partial v_{z}}{\partial r}+\frac{1}{r^{2}}\frac{\partial^{2}v_{z}}{\partial \theta^{2}}+\frac{\partial^{2}v_{z}}{\partial z^{2}}\right)+F_{z} \end{array}$$

where *ρ*, *p* and *ν* are a density, a pressure and dynamic viscosity of liquid in the casing. Since these equations cannot be solved analytically, we apply the boundary conditions listed as follows to convert these equations into linear differential equations so that the circumferential velocity component of the flow *v_θ_* can be obtained [25]:

(a) The flow is not a function of time (steady flow: *∂*/∂t = 0).
(b) The flow is incompressible (*ρ* = const.)
(c) The radial and the *Z*-directional velocity components of the flow are zero (*v*_r_ = 0, *v*_z_ = 0)

Here, we consider the equation of continuity in the cylindrical coordinate system expressed as follows:

(13) $$\frac{1}{r}\frac{\partial \left(v_{r}r\right)}{\partial r}+\frac{1}{r}\frac{\partial v_{\theta }}{\partial \theta }+\frac{\partial v_{z}}{\partial z}=0$$

Applying the boundary condition (c) to the above equation results in $\frac{\partial v_{\theta }}{\partial \theta }=0$, which means that the circumferential velocity component of the flow is constant with respect to *θ*. Regarding this, now we also assume the following boundary conditions:

(d) The circumferential velocity component of the flow is uniform along the *Z*-direction ($\frac{\partial v_{\theta }}{\partial z}=0$)
(e) The pressure distribution is uniform along the circumferential direction ($\frac{\partial p}{\partial \theta }=0$)

By applying these boundary conditions to Equations (10) to (12), the following linear differential equations can be obtained:

(14) $$\frac{{v_{\theta }}^{2}}{r}=-\frac{1}{\rho }\frac{\partial p}{\partial r}$$

(15) $$0=\frac{\partial^{2}v_{\theta }}{\partial r^{2}}+\frac{1}{r}\frac{\partial v_{\theta }}{\partial r}-\frac{v_{\theta }}{r^{2}}$$

(16) $$0=-\frac{1}{\rho }\frac{\partial p}{\partial z}$$

By deriving Equation (14), the following equation can be obtained:

(17) $$v_{\theta }=C_{1}r+C_{2}\frac{1}{r}$$

where *C*_1_ and *C*_2_ are constants. Now the liquid velocity component *v_θ_* at the sidewall of float can be expressed as *aω*_f_(*t*), where *a* is the radius of float. In the same manner, the fluid velocity component *v_θ_* at the sidewall of casing can be expressed as *Rω*_f_(*t*), where *R* is the radius of casing. The constants *C*_1_ and *C*_2_ can therefore be derived as:

(18) $$C_{1}=\frac{1}{a^{2}+R^{2}}\left(a^{2}\omega_{f}(t)-R^{2}\omega_{c}(t)\right)$$

(19) $$C_{2}=\frac{a^{2}R^{2}}{a^{2}+R^{2}}\left(\omega_{c}(t)-\omega_{f}(t)\right)$$

Applying Equations (18) and (19) to Equation (17) gives the following equation:

(20) $$v_{\theta }=\frac{1}{a^{2}-R^{2}}\left\{\left(a^{2}\omega_{f}(t)-R^{2}\omega_{c}(t)\right)r+a^{2}R^{2}\left(-\omega_{f}(t)+\omega_{c}(t)\right)\frac{1}{r}\right\}$$

By applying the above equation to Equation (9), the shear stress *τ_r__θ_* to be generated by the fluid viscosity can be expressed as follows:

(21) $$\begin{array}{cc} \tau_{r\theta } & =\mu r\frac{d}{dr}\left(\frac{1}{a^{2}-R^{2}}\left[\left(a^{2}\omega_{f}(t)-R^{2}\omega_{c}(t)\right)+a^{2}R^{2}\left(-\omega_{f}(t)+\omega_{c}(t)\right)\frac{1}{r^{2}}\right]\right)\\ & =\frac{2\mu a^{2}R^{2}(\omega_{c}(t)-\omega_{f}(t))}{(R^{2}-a^{2})r^{2}} \end{array}$$

The shear stress on the sidewall of float can therefore be obtained from Equation (21) as follows:

(22) $$\tau_{r\theta }{|}_{r=a}=\frac{2\mu R^{2}(\omega_{c}(t)-\omega_{f}(t))}{R^{2}-a^{2}}$$

Since the area of the sidewall of float beneath the liquid surface *S*_side_ = 2π*aT* (*T*: draft of the float), the shear torque *M*_Side_(*t*) to be applied to the sidewalls of the float can be obtained as follows:

(23) $$\begin{array}{cc} M_{\mathrm{side}}(t) & =\tau_{r\theta }\cdot S_{\mathrm{side}}\cdot a\\ & =\frac{4\pi \mu a^{2}R^{2}T(\omega_{c}(t)-\omega_{f}(t))}{R^{2}-a^{2}} \end{array}$$

Finally, By applying the derived torque *M*_Bottom_(*t*) and *M*_side_(*t*) to Equation (1), the following equation can be obtained:

(24) $$\begin{array}{cc} I_{z}\frac{d^{2}\theta_{\mathrm{Yaw}}\left(t\right)}{dt^{2}} & =\pi \mu \left(\frac{a^{4}}{2h}+\frac{4R^{2}a^{2}T}{R^{2}-a^{2}}\right)\left(\omega_{c}\left(t\right)-\omega_{f}\left(t\right)\right)\\ & =\pi \mu K\left(a,h,R,T\right)\left(\omega_{c}\left(t\right)-\omega_{f}\left(t\right)\right) \end{array}$$

Now we include a factor *K*, which is a function containing design parameters of both the reference float and the casing as its arguments. For the reduction of yaw motion of the reference float induced by the angular motion of the casing, as can be seen in the equation, the factor *K* is preferred to be reduced as much as possible. A design study is therefore carried out in this paper to investigate how the design parameters affect *K*. It should be noted that the draft of the float *T* is fixed to be 8.8 mm, regarding the developed prototype sensor described in the following section of this paper.

**Figure 4:**
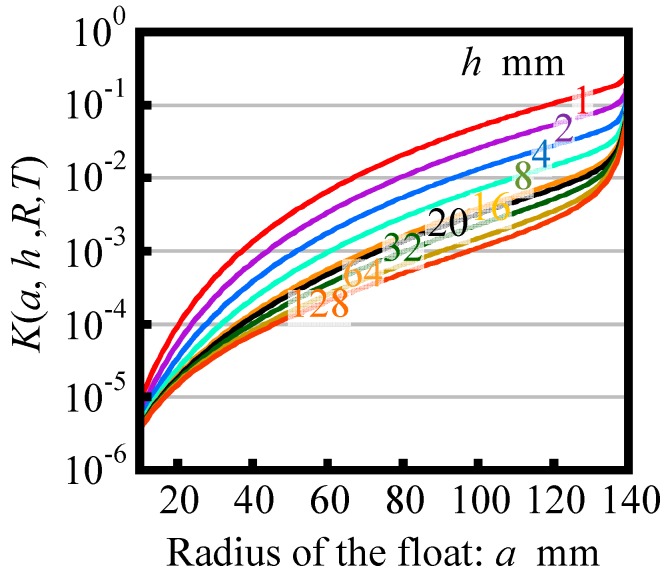
Variation of the factor *K* as a function of the float radius *a* in the case of *R* = 140 mm and *T* = 8.8 mm.

Figure 4 shows the variation of *K* as a function of the radius of reference float *a* in the case of *R* = 140 mm. In the figure, variations of *K* calculated with a distance between the bottom surfaces of float and that of casing *h* ranging from 1 mm to 128 mm are plotted. It should be noted that the ordinate in the graph is scaled logarithmically. As can be seen in the figure, *K* decreases with the decrease of *a*, while its slope continually increases with the decrease of *a* from 135 mm to 10 mm; this result means that the decrease of *a* contributes to reduce the yaw motion of reference float induced by the angular motion of the casing. Meanwhile, *K* is found to dramatically increase with the increase of *a* over 135 mm; this result implies that the increase of *a* reduces a clearance between side surfaces of the float and the casing, resulting in the increase of transmitting torque in between them due to the liquid viscosity. In addition, the increase of *h* is also found to contribute to reduce *K*.

The theoretical analyses described above have revealed that the following designs are expected to reduce the yaw drift of the reference float:

(1) Set the radius of casing *R* as large as possible with respect to the radius of reference float *a*.
(2) Design the radius of float *a* as small as possible.
(3) Set the distance *h* between the bottom surface of the float and that of the casing as large as possible.

Meanwhile, in a practical design of the three-axis inclination sensor, the reference float is required to be floated on a liquid surface. The minimum value of float *a* will therefore be determined while considering not only the buoyancy to be generated by the reference float but also the gravitational force by the masses of scale grating, optical window, and fixing screws. In addition, *R* and *h* will be determined by the size allowed for the three-axis inclination sensor. It should be noted that the influence of *a* on the moment of inertia *I_Z_* of the reference float about the *Z*-axis has not been taken into consideration in this paper for the sake of simplicity, and further detailed investigation is required for the optimization of the design of reference float.

By using the design parameters, the yaw drift of the reference float is simulated. Equation (24) can be modified with respect to the angular velocity of float *ω*_f_(*t*) as follows:

(25) $$\frac{d\omega_{f}(t)}{dt}=\frac{\pi \mu }{I_{z}}\left(\frac{a^{4}}{2h}+\frac{4R^{2}a^{2}T}{R^{2}-a^{2}}\right)(\omega_{c}(t)-\omega_{f}(t))$$

**Figure 5:**
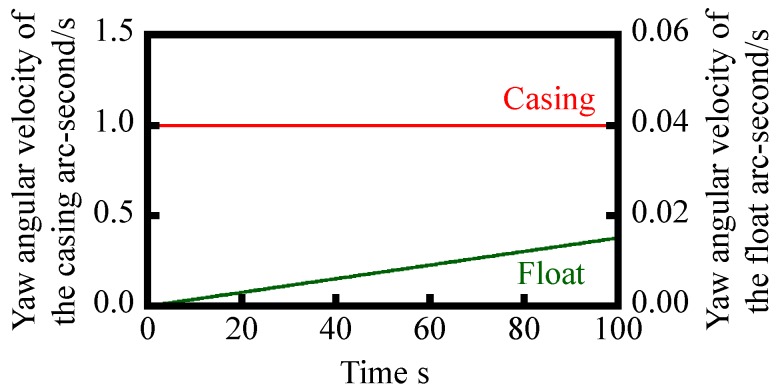
Simulated yaw angular velocity of the reference float due to the yawing given to the casing.

**Figure 6:**
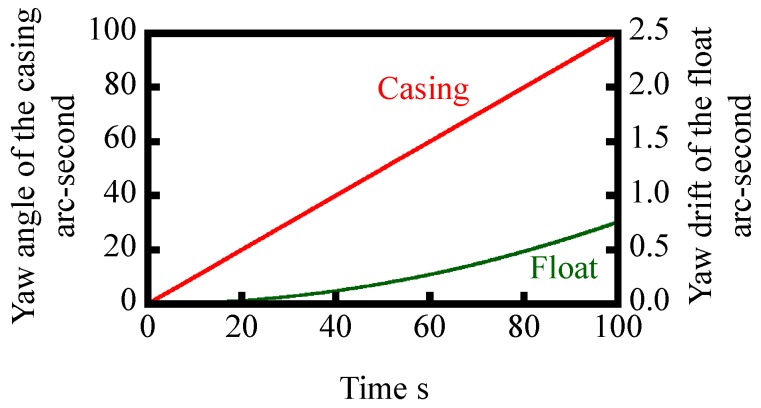
Simulated yaw drift of the reference float due to the yawing given to the casing.

The angular velocity of reference float *ω*_f_(*t*) can be numerically solved by the Runge–Kutta method [26]. In the numerical simulation, a step input with an angular velocity of casing *ω*_c_ of 1 arc-second/s is assumed, while time step is set to be 10 ms. Other parameters for the simulation are summarized in Table 1. Figure 5 shows the result of numerical simulation, in which the change in the angular velocity of the float with respect to time *t* is plotted. By integrating the numerically simulated *ω*_c_(t) shown in Figure 5, an angular displacement of the float *θ*_c_ can be acquired as shown in Figure 6. As can be seen in the figure, the amount of yaw drift increases with the increase of time. However, on the other hand, the amount of yaw drift after 100 s is expected to be less than 1 arc-second; which is comparable or better than the conventional gyros [15].

### 2.3. Modeling of the Pitch and Roll Motions of Inclination Angle Reference Float

**Figure 7:**
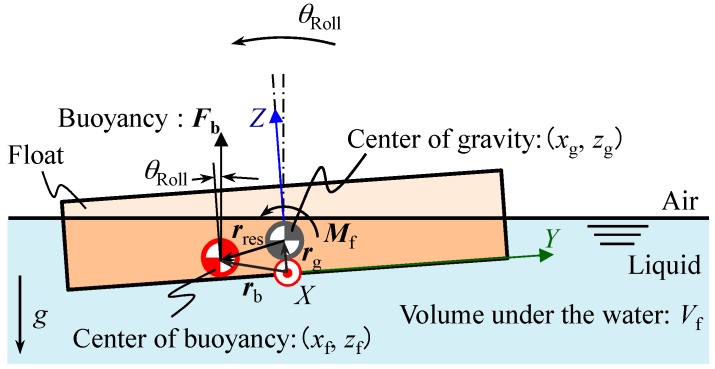
Analytical model for the roll motion of the reference float.

Following the modeling of yaw motion of the reference float, pitch and roll motions of the reference float are also modeled to further investigate the characteristics of the reference float. Since the reference float in this paper is designed to have a disk-like shape symmetrical about the *Z*-axis, the same model can be applied to both the roll and pitch motions of the reference float. Therefore, now we focus on the modeling of the roll motion of the reference float, a schematic of which is shown in Figure 7. 

The equation of angular motion of the reference float can be expressed as follows:

(26) $$I_{X}\frac{d^{2}\theta_{Roll}}{dt^{2}}+c\frac{d\theta_{Roll}}{dt}=M$$

where *I_X_* is the moment of inertia of the reference float about the *X*-axis at the gravitational center, *θ*_Roll_ is the roll angle, *c* is the viscosity coefficient of the liquid, and *M* is a torque applied to the reference float. The buoyancy **F***_b_* can be expressed as follows:

(27) Fb=[0, ρgVfsinθRoll, ρgVfcosθRoll]t

where *ρ* is the liquid density, *g* is the gravity, and *V*_f_ is the volume of reference float beneath the liquid surface. The point of application of buoyancy (*y*_f_, *z*_f_) coincides with a center of the volume beneath the liquid surface, and can be expressed as follows:

(28) $$y_{f}=\frac{\int xdV}{\int dV},\;z_{f}=\frac{\int zdV}{\int dV}$$

**Figure 8:**
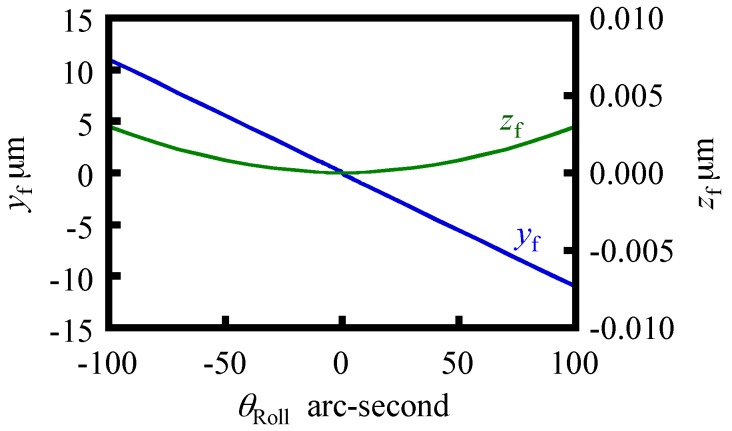
Variation of the point of application of buoyancy.

In this paper, the point of application of buoyancy (*y*_f_, *z*_f_) is calculated by using a function in a three-dimensional CAD software. The parameters employed in the simulation are summarized in Table 2. Figure 8 shows variations of *y*_f_ and *z*_f_ with respect to *θ*_Roll_ ranging from −100 arc-seconds to 100 arc-seconds in the case of *T* = 10 mm and *a* = 30 mm. 

As can be seen in the figure, *y*_f_ and *z*_f_ can be approximated by a linear function and a second-order polynomial functions, respectively, with respect to the change in *θ*_Roll_. Since *z*_f_ is much smaller than *y*_f_, now we assume *z*_f_ to be zero for the sake of simplicity. Under the condition of *T* = 10 mm and *a* = 30 mm, a gradient of the approximated line of *y*_f_ is evaluated to be −0.1091 mm/arc-second. Now a positional vector ***r***_res_ pointing toward the gravitational center (*y*_g_, *z*_g_) of the reference float from the center of buoyancy can be expressed as follows:

(29) rres=rf−rg=[0, yf−yg, zf−zg]t

Rotational moment ***M***_f_ to be applied to the reference float due to the buoyancy can therefore be expressed as follows:

(30) Mf=rres×Fb=[0, yf−yg, zf−zg]t×[0, ρgVfsinθRoll, ρgVfcosθRoll]t=[(yf−yg)ρgVfcosθRoll−(zf−zg)ρgVfsinθRoll, 0, 0]t=[ρgVf(yfcosθRoll+zgsinθRoll), 0, 0]t

where *y*_g_ is also assumed to be zero due to the symmetrical shape of the reference float. Furthermore, since the measurement ranges of the developed inclination sensor for both the pitch and roll angle will be designed to be smaller than ±50 arc-seconds, by approximating cos*θ*_Roll_ ≈ 1 and sin*θ*_Roll_ ≈ *θ*_Roll,_ ***M***_f_ in Equation (30) can be simplified as follows:

(31) Mf=[ρgVf(α+zg)θRoll, 0, 0]t

As can be seen in the equation, the moment will be generated about the *X*-axis in Figure 7. The equation of roll motion of the reference float can be obtained as follows by applying **M***_f_* in Equation (31) to Equation (26):

(32) $$I_{X}\frac{d^{2}\theta_{Roll}}{dt^{2}}+c\frac{d\theta_{Roll}}{dt}=\rho gV_{f}(\alpha +z_{g})\theta_{Roll}$$

In the same manner, the equation of pitch motion of the reference float can be obtained. It should be noted that the natural frequencies of pitch and roll motions of the reference float *f*_p_ and *f*_r_, respectively, can be derived from Equation (32) as follows:

(33) $$f_{r}=\frac{1}{2\pi }\sqrt{\frac{-\rho gV_{f}\left(\alpha +z_{g}\right)}{I_{X}}},f_{p}=\frac{1}{2\pi }\sqrt{\frac{-\rho gV_{f}\left(\alpha +z_{g}\right)}{I_{Y}}}$$

Since *α* < 0, *f*_p_ can exist over the region of *z*_g_ < −*α*. Attention should be paid when designing the reference float so that the frequency bandwidth of the three-axis inclination sensor will not overlap the natural frequency *f*_p_.

## 3. Design of the Three-Axis Inclination Sensor

### 3.1. An Optical Sensor Head

**Figure 9:**
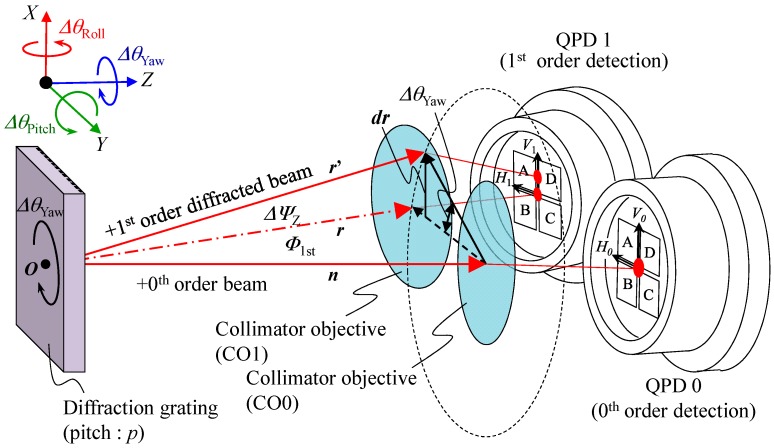
Principle of the three-axis laser autocollimation.

In the proposed three-axis inclination sensor, an optical sensor head of the angle sensor based on the three-axis laser autocollimation [16] is employed, while a scale grating having linear pattern structures with a constant period *p* is employed as a reflector and is mounted on the float. Figure 9 shows a schematic of the geometric relationship between the optical sensor head and the scale grating. As can be seen in the figure, a measurement laser beam made incident to the scale grating will generate zeroth-order diffracted beam, and first-order diffracted beams in positive and negative directions. It should be noted that the negative first-order diffracted beam is omitted in the figure for the sake of clarity.

Two-dimensional tilt angles of the scale grating about the *X*- and/or *Y*-axes with respect to the optical sensor head can be measured based on the two-axis laser autocollimation [17]. By detecting displacements of a focused laser beam Δ*v*_0_ and Δ*h*_0_ along the *V*_0_- and *H*_0_-axes on the quadrant photo diode (QPD) for the detection of zeroth-order diffracted beam (QPD0), respectively, the angular displacements of the scale grating Δ*θ*_Roll_ and Δ*θ*_Pitch_ about the *X*- and *Y*-axes, respectively, can be obtained as follows [27]:

(34) $$\Delta \theta_{\mathrm{Roll}}=\frac{1}{2}\arctan\frac{\Delta h_{0}}{f},\;\Delta \theta_{\mathrm{Pitch}}=\frac{1}{2}\arctan\frac{\Delta v_{0}}{f}$$

where *f* is a focal length of the collimator objective (CO0). Meanwhile, the tilt angle of scale grating about the *Z*-axis does not affect the position of focused laser beam on QPD0; this fact means that the change in yaw angle of the scale grating cannot be detected by the QPD0. However, on the other hand, a QPD for the positive first-order diffracted beam (QPD1) can detect the change in yaw angle of the scale grating. Now the diffraction angle of the first-order diffracted beam *ϕ*_1st_ with respect to the normal of the scale grating can be expressed as follows [28]:

(35) ϕ1st=arcsin(λp)

where *λ* is the wavelength of laser beam made incident to the scale grating. Now we denote a directional vector of the zeroth-order diffracted beam as ***n***. Also, we denote the directional vectors of the positive first-order diffracted beam with the yaw angle of zero and Δ*θ*_Yaw_ as ***r*** and ***r***’, respectively. According to the geometric relationship, the following equation can be obtained [16]:

(36) $$r^{\prime }=r\cos\Delta \theta_{Yaw}+n\left(n\cdot r\right)\left[1-\cos\Delta \theta_{Yaw}\right]+\left(n\times r\right)\sin\Delta \theta_{Yaw}$$

On the assumption that Δ*θ*_Yaw_ is small, denoting the ***r***’ − ***r*** = ***dr***, Equation (36) can be modified as follows:

(37) $$\mathrm{dr}=r^{\prime }-r≅\left(n\times r\right)\Delta \theta_{Yaw}$$

The angle Δ*ψ*_Z_ between the vectors ***r*** and ***r***’ can be expressed by using ***r*** and ***dr*** as follows:

(38) $$\Delta \psi_{Z}≅\frac{\left\|\mathrm{dr}\right\|}{\left\|r\right\|}=\Delta \theta_{Yaw}\sin\phi_{1st}$$

Since Δ*ψ*_Z_ is the angle of incidence of the first-order diffracted beam with respect to the collimator objective 1 (CO1), according to the principle of the laser autocollimation, the displacement Δ*v*_1_Yawing_ of focused first-order diffracted beam on the QPD1 along the *V*_1_-axis due to Δ*θ*_Yaw_ can be obtained as follows:

(39) $$\Delta v_{1\_Yawing}=f\tan\Delta \psi_{Z}≅f\Delta \psi_{Z}=\frac{f\lambda \Delta \theta_{Yaw}}{p}$$

By modifying the above equation, the change in yaw angle of the scale grating about the *Z*-axis Δ*θ*_Yaw_ can be expressed as follows:

(40) $$\Delta \theta_{Yaw}=\frac{p}{f\lambda }\Delta v_{1\_Yawing}$$

It should be noted that the first-order diffracted beam focused on the QPD1 could be moved along the *V*_1_-axis not only by Δ*θ*_Yaw_ but also by Δ*θ*_Pitch_. The change in yaw angle of the scale gratings can therefore be obtained from the following equations:

(41) $$\Delta \theta_{Yaw}=\frac{p}{f\lambda }\Delta v_{1\_Yawing}=\frac{p}{f\lambda }\left(\Delta v_{1}-\Delta v_{1\_Pitching}\right)=\frac{p}{f\lambda }\left(\Delta v_{1}-\Delta v_{0}\right)$$

**Figure 10:**
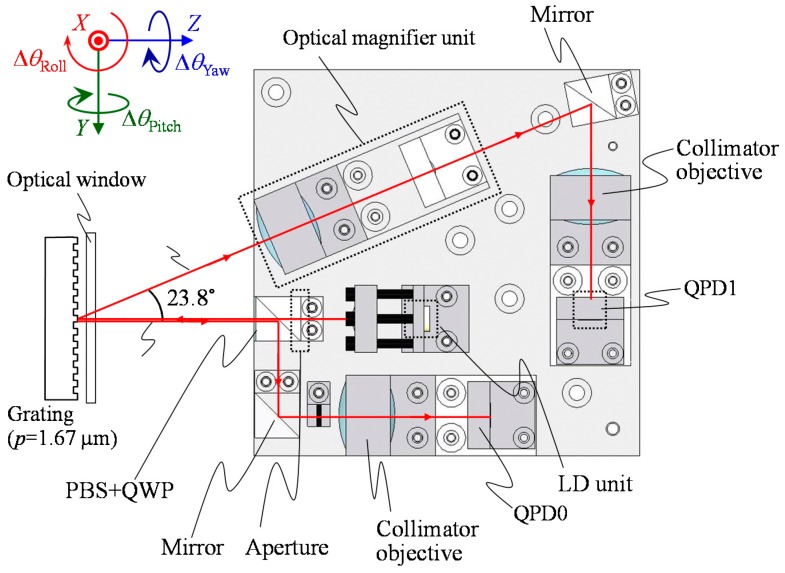
A schematic design of the optical sensor head.

Figure 10 shows an optical design of the optical sensor head for the three-axis inclination sensor. The detailed design parameters of the optical sensor head are summarized in Table 3. As the light source for the optical sensor head, a laser diode with a wavelength of 685 nm was employed. As the two-dimensional photodetectors, quadrant photodiodes with insensitive gaps of 5 µm (S6795, Hamamatsu Photonics, Hamamatsu, Japan) and 10 µm (Hamamatsu Photonics S1651-03) were employed to detect the zeroth and first-order diffracted beams, respectively. It should be noted that the widths of insensitive gaps of the QPDs were small enough to detect the laser beam focused by the collimator objectives. All the optical components were assembled in a home-made optical sensor base designed in a size of within 100 mm × 100 mm. It should be noted that the influence of the earth curvature on the evaluation of pitch and roll angles cannot be neglected [29] in the case of evaluating stage systems having a long travel range in the *X*- and/or *Y*-directions, and needs to be corrected when further better measurement accuracy is required.

### 3.2. Reference Float

**Figure 11:**
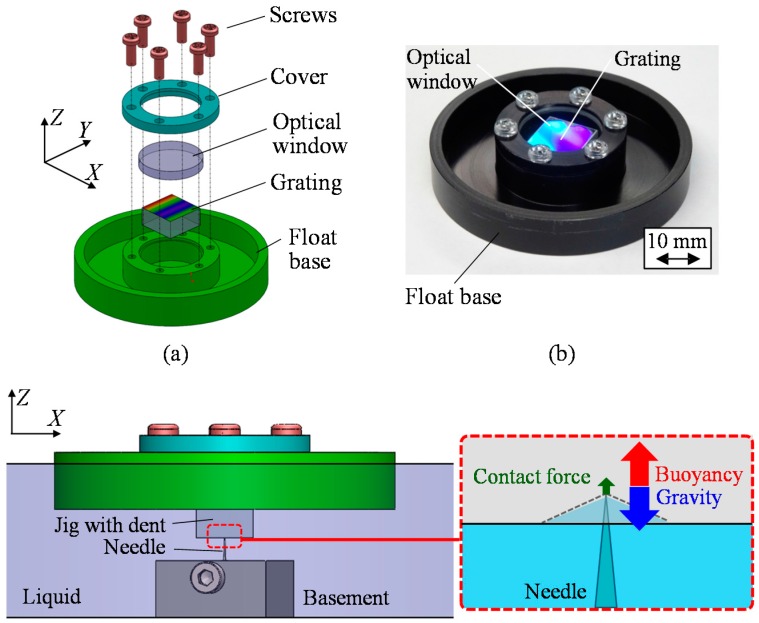
Design of the reference float: (**a**) Exploded perspective view; (**b**) A photograph; (**c**) The floating unit and a supporting needle beneath the liquid surface.

Figure 11a shows a schematic of the designed reference float, which is composed of a float, a scale grating, an optical window, a cover and screws. Regarding the simulation results described in the previous section of this paper, aiming to reduce the yaw drift, the reference float is designed to be as compact as possible. In addition, attentions have also been paid to suppress the total weight of the reference float by using materials with light weights for each of the components. The detailed design parameters of the reference float are summarized in Table 4. The design parameters of the reference float were determined mainly by the size of scale grating. From Equation (33), the natural frequency *f_r_* and *f_p_* of the reference float were evaluated to be 6.01 Hz. It should be noted that higher *f_r_* and *f_p_* can be obtained by decreasing *I*_X_ and *I*_Y_ with a design modification of the reference float. Meanwhile, the design modification for decreasing *I_X_* and *I_Y_* could lead to the increase of *I_Z_*, resulting in the increase of yaw drift of the reference float. Attentions should therefore be paid to carry out the design optimization of the reference float, which will be carried out in future work as the next step of the research. Figure 11b shows a photograph of the developed reference float, on which a scale grating is mounted.

In the proposed three-axis inclination sensor, the reference float is required to be held stationary along the *X*-, *Y*- and *Z*-directions while allowing its rotational motion about each axis. A supporting needle is therefore introduced in the system in such a way that the needle is fixed at the middle of the bottom surface of the casing, while a jig with dent is placed beneath the reference float as shown in Figure 11c; a tip of the needle is in a point contact with the bottom surface of the reference float through the jig, which makes the reference float possible to be held stationary along the *X*-, *Y*- and *Z*-directions, while allowing the rotational motion about each axis.

### 3.3. System Integration

**Figure 12:**
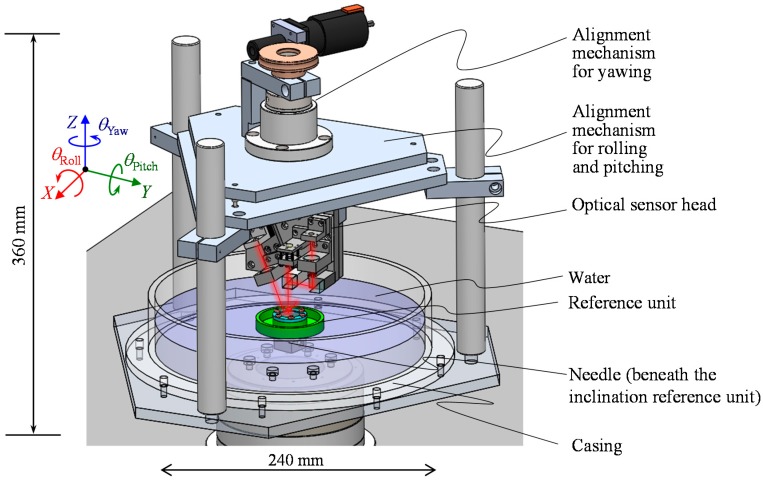
A schematic of the developed three-axis inclination sensor.

The developed sensor head and the reference float are integrated into the three-axis inclination sensor, a schematic of which is shown in Figure 12. The three-axis inclination sensor is composed of the reference float and a three-axis angle detection unit, which consists of the casing with an inner diameter of 240 mm filled with water, the optical sensor head and its alignment mechanisms. The casing is rigidly connected to the optical sensor head through poles and the alignment mechanisms. The reference float is made to float on the water surface, while it is point-supported at its bottom surface by a needle fixed on the bottom surface of the casing. All the sensor system has been constructed in a diameter of approximately 300 mm and a height of 360 mm.

## 4. Evaluation of the Basic Characteristics of the Developed Three-Axis Inclination Sensor

**Figure 13:**
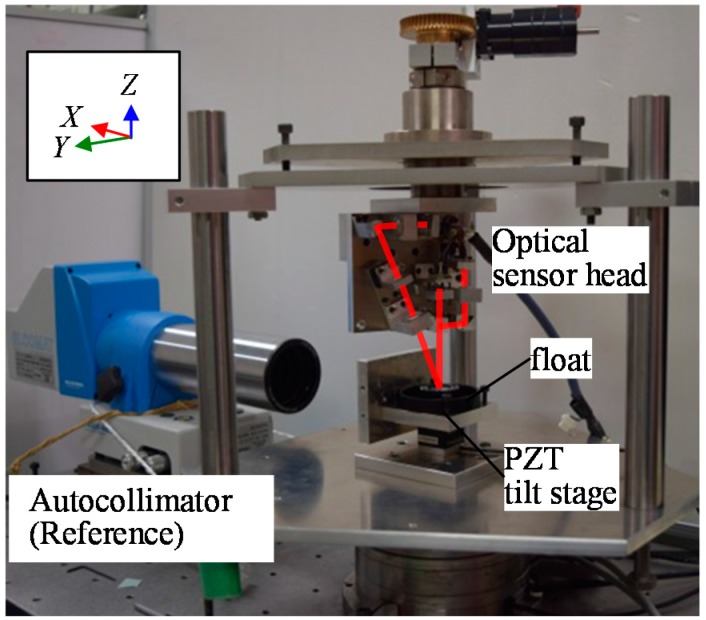
A schematic of the experimental setup.

**Figure 14:**
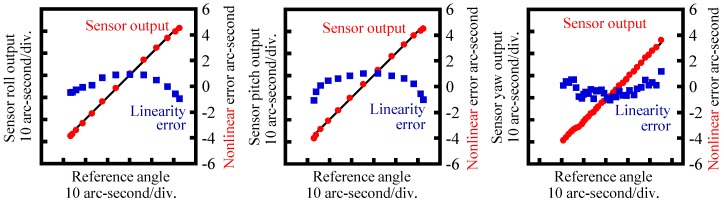
Verification result of the angle sensor output.

By using the developed prototype three-axis inclination sensor, the basic characteristics of the proposed method have been verified in experiments. First, the static characteristics of the developed optical sensor head were evaluated. Figure 13 shows a photograph of the experimental setup. To avoid the influence of instability of the reference float being floated on the liquid surface, the reference float was mounted on a stage system fixed on the bottom surface of the casing in which the water was drained. To apply tilt angles to the reference float, a two-axis precision PZT tilt stage was employed as the stage system. The tilt angles of the reference float about the *Y*- and *Z*-axes (the pitch and yaw angles, respectively) were also measured by using a commercial two-axis laser autocollimator (Elcomat 3000, Möller-Wedel, Germany), which was employed in the setup as a reference. A mirror was mounted on a side face of the PZT tilt stage as a reflector for the commercial laser autocollimator. It should be noted that the commercial autocollimator in the setup shown in Figure 13 could not measure roll angle (tilt angle about the *X*-axis). However, this could be measured by placing the commercial autocollimator in such a way that the measurement axis of the commercial autocollimator coincided with the *Y*-axis, while re-mounting the reflector on the other sideface of the PZT tilt stage. In the experiments, tilt angles were applied to the reference float by the PZT tilt stage, while measuring the tilt angles with both the developed optical sensor head and the commercial laser autocollimator. Figure 14a–c show the measured angular displacements about the *X*-, *Y*- and *Z*-axes, respectively. In the figures, the horizontal axes show the tilt angles measured by the commercial laser autocollimator, while the vertical axes show the tilt angles measured by the developed optical sensor head. Linearity errors are also shown in the figures. As can be seen in the figures, good correlations can be found between the outputs from the developed sensor and those from the commercial laser autocollimator. The nonlinear errors of the roll, pitch and yaw measurement were evaluated to be ±0.93 arc-second, ±1.05 arc-seconds and ±1.20 arc-seconds, respectively, over a range of approximately 50 arc-seconds. These values are comparable to the results shown in [16]. It should be noted that the sensitivity of yaw angle measurement was low compared with those of pitch and roll angle measurement; which is mainly due to the different principle of angle detection, as can be seen in Equations (35) and (41). It should also be noted that the calibration and sensitivity verification of the pitch and roll measurement of the developed three-axis inclination sensor can be carried out by the procedure described above, while those for the yaw measurement can be carried out by mounting the three-axis inclination sensor onto a precision spindle equipped with precision rotary encoder.

**Figure 15:**
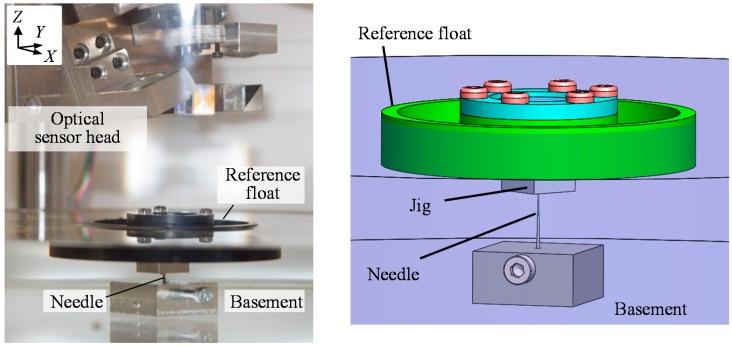
The reference float floating on water surface with the point-support of a needle.

**Figure 16:**
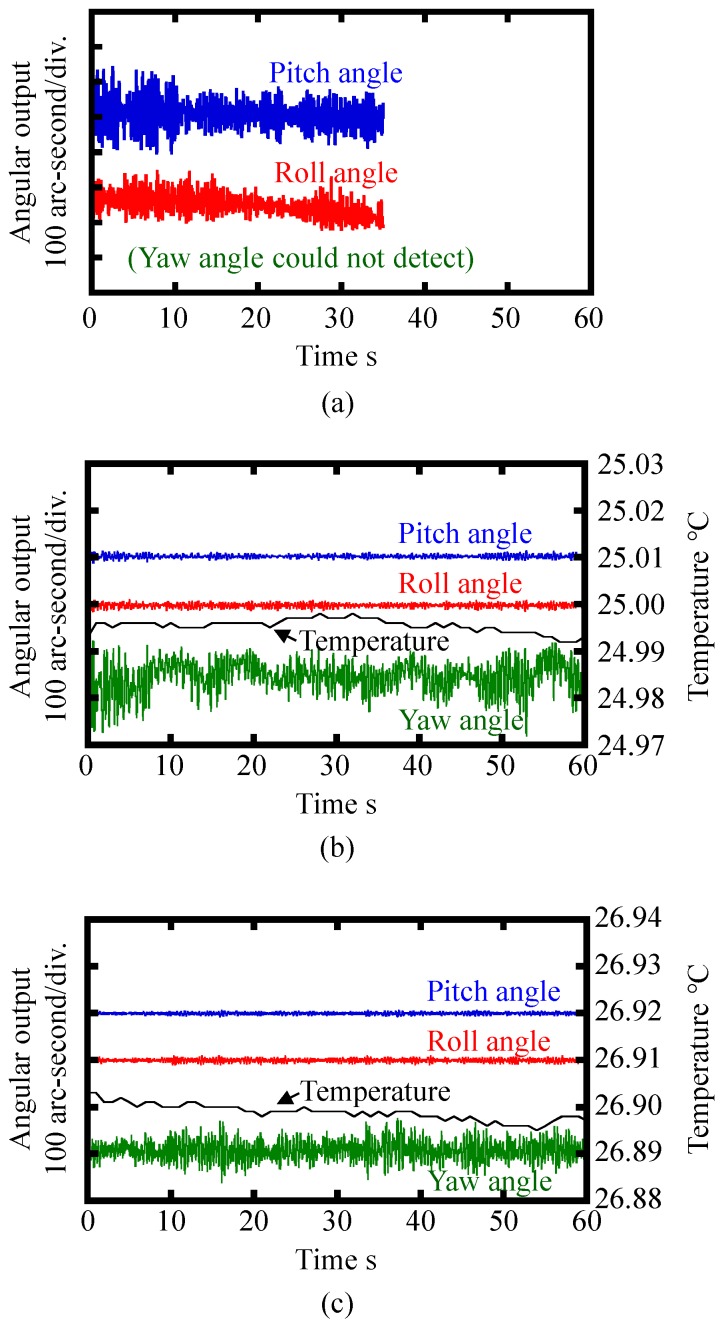
Stability of the three-axis inclination sensor output: (**a**) Without the fixing needle; (**b**) With the fixing needle while not using the cover plate on the liquid surface; (**c**) With the fixing needle and the cover plate on the liquid surface.

After the verification of static characteristics of the developed optical sensor head, stability of the three-axis inclination sensor was verified in experiments. Figure 15 shows a setup for the experiment. At first, an effect of the point-support by the needle was verified. The reference float was made to float on the water surface in the casing without the support of the needle, and the variations of the three-axis outputs from the optical sensor head were evaluated. Figure 16a shows the results. As can be seen in the figure, it was difficult to continue the experiment due to the instability of the reference float on the water surface. Then, the reference float was point-supported by the needle fixed on the bottom surface of the float, and the experiment was carried out again. Figure 16b shows the result. As can be seen in the figure, stable measurement of the pitch and roll angle was realized with the enhancement of the point-support by the needle. Furthermore, yaw angle measurement, which could not be carried out when the reference float was made to freely float on the water surface, was successfully carried out with the help of point-support by the needle. It should be noted that the water level in the casing was determined to be 28 mm by trial and error throughout the experiments to adjust the contact force between the reference float and needle for the optimization of sensor stability. The standard deviations of the pitch, roll and yaw angle output during the period of 60 s were evaluated to be 3.4, 3.9 and 32.9 arc-seconds, respectively. A relatively large deviation of the yaw angle output was considered to be mainly due to lower yaw measurement sensitivity and instability induced by standing waves in water. It should be noted that the results acquired by the developed prototype were not as good as expected from the principle of three-axis laser autocollimation [16] due to the instability of the reference float. Signal processing techniques such as low-pass filtering have a possibility of decreasing the resolution to the 1 arc-second level in the applications where a low frequency bandwidth of the three-axis inclination sensor could be accepted.

Since it is difficult to eliminate all the external disturbances which result in the occurrence of the standing waves in water, in this paper, a ring-shaped cover plate was introduced into the three-axis inclination sensor to suppress the influences of standing waves. The cover plate with outer and inner diameters of 90 and 230 mm, respectively, and a thickness of 5 mm was made to float on the water surface. The ring-shaped cover was made to float freely on the liquid surface. The inner and outer diameters of the cover were determined in such a way that the collision between the inner edge of the cover and the reference float could be avoided, while allowing the cover to contact with the casing. Figure 16c shows the measured stability of the three-axis inclination sensor with the cover plate. As can be seen in the figure, deviation of pitch, roll and yaw angle output during the period of 60 s were reduced to be 1.8 arc-seconds, 2.9 arc-seconds and 18.6 arc-seconds, respectively; especially a low-frequency component in the yaw angle output was reduced. The introduced cover plate was considered to work as a damper for reducing the standing waves in water, resulting in the reduction of deviation of yaw angle output.

**Figure 17:**
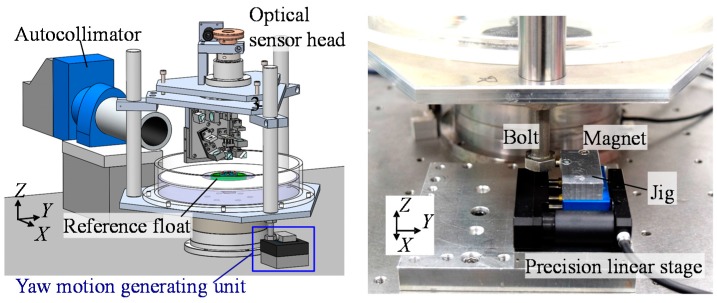
A schematic of the experimental setup for evaluation of the yaw drift.

The experiment was extended to address another main concern of the developed three-axis inclination sensor: yaw drift induced by the liquid flow in the casing. Figure 17 shows a schematic of the experimental setup. The three-axis inclination sensor was mounted on a rotary stage that could freely be rotated about the *Z*-axis. Meanwhile, due to its heavy weight, the rotary stage could not rotate the three-axis inclination sensor with its actuator. Therefore, an external linear slide was employed to give an angular displacement to the inclination sensor about the *Z*-axis. The external linear slide was placed beneath the casing, and was made to push a rod connecting the casing and the optical sensor head. It should be noted that a magnet was embedded to the top plate of the linear slide so that the three-axis inclination sensor could be held stationary after giving the angular displacement about the *Z*-axis to the sensor. As can be seen in Figure 17, a commercial autocollimator was employed as a reference sensor to verify the measurement result by the developed three-axis inclination sensor.

**Figure 18:**
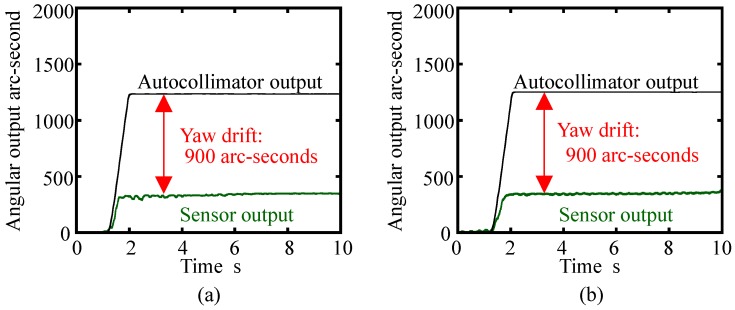
Yawing of the reference float measured by the developed inclination sensor: (**a**) Without the ring-shaped cover plate; (**b**) With the ring-shaped cover plate.

Figure 18a shows the result without the cover plate. In the figure, both the angular output from the commercial autocollimator and the three-axis inclination sensor were plotted. As can be seen in the figure, the developed three-axis inclination sensor successfully detected the given yaw motion. According to the output from the commercial autocollimator, an angular displacement of approximately 1200 arc-seconds was applied to the three-axis inclination sensor. Meanwhile, the output from the inclination sensor was approximately 300 arc-seconds; this result implies that the yaw drift due to the angular motion generated by the linear slide was approximately 900 arc-seconds, which was larger than expected from the simulation described in the previous section of this paper. This result implies that the reference float is sensitive to the step input. A possible root-cause of these results, which were different from the ones estimated in the analytical calculations, is the frictional force between the reference float and the tip of needle. The change in fluid behavior could also induce the shear effect between the reference float and the casing. Meanwhile, on the other hand, variation of the inclination sensor output after giving the step input to the float was quite small. In addition, the sensor output was quite stable: this result indicates the possibility of the application of this sensor for the evaluation of a precision linear slide, which has small angular error motion about each axis. Figure 18b shows the result with the cover plate. As can be seen in the figure, it was verified that the existence of the cover plate did not affect the yaw drift of the reference float.

It should be noted that, in this paper, attention has been paid to verifying the possibility of the proposed three-axis inclination sensor, and only basic experiments have been carried out to demonstrate the feasibility of the developed prototype sensor. Further detailed investigation on the static and dynamic characteristics of the three-axis inclination sensor with an optimized setup, influence of the frictional force between the supporting needle and the reference float, and the application to the evaluation of angular error motions of precision linear slides after the improvement of measurement resolution with the enhancement of signal processing techniques such as low-pass filtering, will be carried out in future work.

## 5. Conclusions

In this paper, a new three-axis inclination sensor concept, which is composed of an optical sensor head and a reference float capable of carrying out simultaneous measurement of three-axis tilt angles of a measurement target, has been proposed. In the proposed three-axis inclination sensor, the reference float, which is made to float on a water surface, has been employed as a reference for the detection of three-axis tilt angles, while the optical sensor head based on the three-axis laser autocollimation has been employed to detect its relative angular displacement with respect to the reference float. Meanwhile, stability of the reference float is one of the most important issues to be addressed for the proposed concept of the three-axis inclination sensor. Therefore, as the first step of this research, establishments of simplified kinematic models of the reference float for angular motion about three axes and theoretical analyses based on the established kinematic models have been carried out to search for the possibility of realizing the proposed concept of the three-axis inclination sensor. Results of the analytical calculations based on the established kinematic model have shown that the yaw drift of the reference float due to the angular motion of the casing in the inclination sensor, can be suppressed to be less than 1 arc-second for 60 s by optimizing the geometrical designs of the reference float and a casing in the three-axis inclination sensor. Furthermore, a prototype of the three-axis inclination sensor has been designed and constructed to search for the possibility of the proposed concept. To carry out measurement by the developed prototype, the reference float has to be placed on the water in a stable state. For stabilizing the reference float on the water surface while not restricting its angular motions about the three axes, a unique point-supporting mechanism with a tiny needle has been introduced to the sensor. Some basic experiments have been carried out by the developed sensor prototype, and an effect of the point-supporting mechanism has successfully been demonstrated. In addition, with the enhancement of a ring-shaped cover plate, which has been integrated into the sensor for the purpose of suppressing standing waves in water, stable simultaneous three-axis angular motion measurement has been realized.

It should be noted that this paper has focused on the verification of the possibility of proposed concept of the three-axis inclination sensor. Further detailed investigation on the influence of the change in the moment of inertia *I_z_* of the reference float, verification tests such as evaluation of static and dynamic characteristics of the proposed sensor including analysis on its measurement uncertainty will be carried out in future work, as well as further improvement of the resolution with the enhancement of signal processing techniques such as low-pass filtering.

## Figures and Tables

**Table 1:** Parameters for analytical calculations.

Component	Parameters	Symbol	Value
Float	Radius	*a*	30 mm
	Draft	*T*	8 mm
	Moment of inertia about the Z-axis	*I_z_*	9.02 × 10^−4^ kg m^2^
Casing	Radius	*R*	120 mm
	Water level	*h*	80 mm
Fluid	Type	-	Water
	Viscosity	*μ*	0.89 × 10^−3^ Pa s
	Density	*ρ*	1.00 × 10^3^ kg/m^3^

**Table 2:** Parameters for simulation.

Parameters	Symbol	Value
Moment of inertia of the float about the X-axis	*I_X_*	5.17 × 10^−4^ kg m^2^
Sensitivity coefficient	*α*	−0.0281 m/rad
Gravity	*g*	9.80 m/s^2^

**Table 3:** Parameters of the three-axis angle sensor.

Parameters	Value
Light wavelength	685 nm
Aperture diameter	2 mm
Grating period	1.67 μm
Focal length of the collimator objective	25.4 mm

**Table 4:** Parameters of the reference float.

Parameters	Symbol	Value
Radius	*a*	30 mm
Thickness		10 mm
Draft	*T*	8.8 mm
mass		24.4 × 10^−3^ kg
Moment of inertia about the Z-axis	*I_z_*	9.02 × 10^−4^ kg m^2^
Moment of inertia about the X/Y-axis	*I_X_/I_Y_*	5.17 × 10^−4^ kg m^2^
Material	-	Polyoxymethylene
Density	*ρ*	1390 kg/m^3^
